# Evaluating the effect of infographics on public recall, sentiment and willingness to use face masks during the COVID-19 pandemic: a randomised internet-based questionnaire study

**DOI:** 10.1186/s12889-021-10356-0

**Published:** 2021-02-17

**Authors:** Mark Egan, Amish Acharya, Viknesh Sounderajah, Yihan Xu, Abigail Mottershaw, Rosie Phillips, Hutan Ashrafian, Ara Darzi

**Affiliations:** 1 The Behavioural Insights Team (BIT), 2nd Floor, 4 Matthew Parker Street, London, SW1H 9NP UK; 2 Institute of Global Health Innovation, Department of Surgery & Cancer, Imperial College London , 10th Floor Queen Elizabeth Queen Mother Building, St Mary’s Hospital, London, W2 1NY UK

**Keywords:** Masks, COVID-19, Personal protective equipment, Audiovisual aids, Health behavior

## Abstract

**Background:**

The use of face masks remains contentious, with international variation in practice. Their prevalence in the UK, is likely to increase due to new legislation. Clear information regarding the appropriate use of masks is needed, to ensure compliance with policies to reduce transmission of COVID-19. We aimed to assess the impact of visual representations of guidance, or infographics, upon the knowledge of appropriate face mask usage in a representative UK cohort.

**Methods:**

Adult patients were recruited to this randomised internet-based questionnaire study during the 12–14 May 2020 from across the UK. Respondents viewed one of five public health stimuli regarding the use of face masks, or no stimulus. The groups accessed aids by the European Centre for Disease Control (EUCDC), World Health Organisation (WHO), Singaporean Ministry of Health (SMOH), text from the UK government (UK Gov), or an infographic designed by the Behavioural Insights Team (BIT). The primary outcome was to evaluate the effect of each infographic upon participants’ recall of face mask technique, sentiments and willingness to wear a face covering. Secondary outcomes included the effect of symptomology and socio-demographic factors.

**Results:**

4099 respondents were randomised (1009 control, 628 EUCDC, 526 WHO, 639 SMOH, 661 UKGOV and 606 BIT). Stimuli from the WHO, SMOH and BIT demonstrated significantly higher average recall scores compared to the controls (7.40 v. 7.38 v. 7.34 v. 6.97, *P* < 0.001). BIT’s stimulus led to the highest confidence about mask-wearing (87%). Only 48.2% of the cohort felt stimuli reduced anxiety about COVID-19. However, willingness to use a mask was high, (range 84 to 88%).

**Conclusions:**

To ensure the appropriate use of masks, as mandated by UK law, guidance must provide sufficient information, yet remain understandable. Infographics can aid the recall of correct mask techniques by highlighting salient steps and reducing cognitive burden. They have also demonstrated greater trustworthiness than text-only guidance. The effect of infographics upon COVID-19-related anxiety was poor, and they should be further developed to address this sentiment. A willingness to wear face masks has, however, been demonstrated.

**Supplementary Information:**

The online version contains supplementary material available at 10.1186/s12889-021-10356-0.

## Background

The COVID-19 pandemic has had an unprecedented impact upon communities, with a significant number of cases and deaths worldwide [1]. Emergency lockdown and social distancing measures have become commonplace amongst populations globally, in an attempt to control the rates of infection [2, 3]. With vaccines and curative treatments yet to be developed, infection control measures remain at the forefront of health policy.

Unlike other preventative measures, the use of facemasks has remained controversial [4]. Whilst countries such as Singapore mandated the use of masks in April, others including the US and UK have been slower at adopting this approach [5, 6]. At the time of Singaporean legislation, only 13% of Britons admitted wearing a face mask in public places, compared to 50% in Thailand, and 80 to 90% of people in China and Italy [7, 8]. As of the 24th July 2020 in England however, it has become a legal requirement to wear a face covering on public transport and in many indoor locations including shops, banks, and post offices. Law enforcement agencies are even able to issue fines of up to £100 to those who fail to comply [9]. According to a YouGov tracker, following these regulations there was a surge in British respondents reporting wearing masks in public, but overall the level was low at 57% [7] This cultural schism in the public perception of and legislation mandating face coverings has also been seen in other European countries. In a study by Wang et al. despite Polish participants reporting significantly higher rates of recent fever or breathing difficulties, hospitalisation and need for consultation with a doctor, only 35% utilised precautionary measures. In contrast, reported face mask use was 60% higher amongst Chinese respondents, who also demonstrated significantly lower rates of healthcare utilisation or symptomology [10].

In spite of this, there is growing evidence as to the use of face coverings to mitigate transmission [11]. Current evidence suggests that COVID-19 is predominantly spread through respiratory secretions. Although there is also growing concern regarding the potential for spread through inhalation of airborne microdroplets [12]. The use of masks, however, has been shown to reduce contraction of acute respiratory viruses by 46% [13]. In addition, no human Coronavirus RNA was detectable in samples of air droplets or aerosols of those wearing masks [14]. Furthermore, in a retrospective analysis of confirmed COVID-19 cases in Beijing, the risk of transmission was 79% lower in households where one or more family members wore a mask, compared to those where no one did [15].

The greater understanding of modes of transmission, evidence of efficacy and subsequent national guidance, has therefore led to an inevitable increase in the support and prevalence of face masks. In fact, polling of 3208 adults, conducted by the Behavioural Insights Team (BIT) from late March to early May 2020, found that support for the mandatory use of masks in public, increased by 21 percentage points over this period. This increase in support was most notable amongst those aged 55 and older [16]. Despite this, usage is not yet at the level of other countries, in which face covering may conform with normative behaviours. It is, therefore, even more imperative that there is clear guidance on the correct application of coverings, given improper use is likely to negate the benefit of the mask, and potentially risk harm. This poses a substantial challenge for behavioural scientists and policy makers alike. Several differing resources have produced visual guidance to aid the public, however the impact of these stimuli has not been critically appraised [17–21]. Moreover, understanding the impact these visual aids have upon the willingness of members of public to use masks, is key.

The aim of this evaluation was to discern the impact on public knowledge, sentiment and willingness to use face masks during the COVID-19 pandemic, of five widespread online infographics or guidance pages.

## Methods

**Fig. 1:**
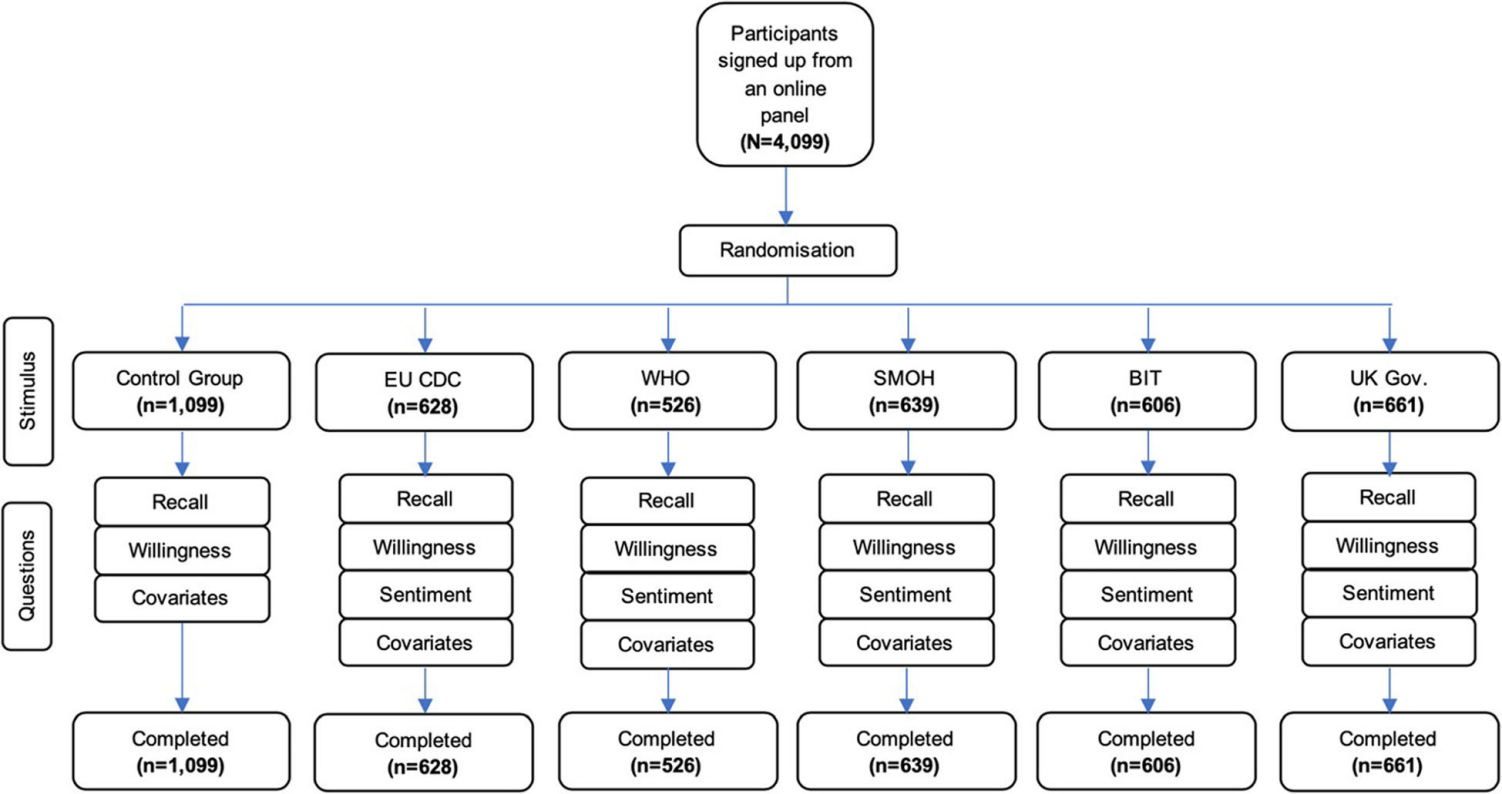
Diagram to demonstrate participant flow throughout the study

The study was undertaken to evaluate the understanding of public health guidance on a representative sample of the adult UK population. It was conducted between the 12th to 14th May 2020. Participants were randomly allocated by means of computer-generated random number generator to one of five trial arms, each of whom were asked to review a differing infographic or visual stimulus describing how to wear a face mask, see Fig. 1.

**Fig. 2:**
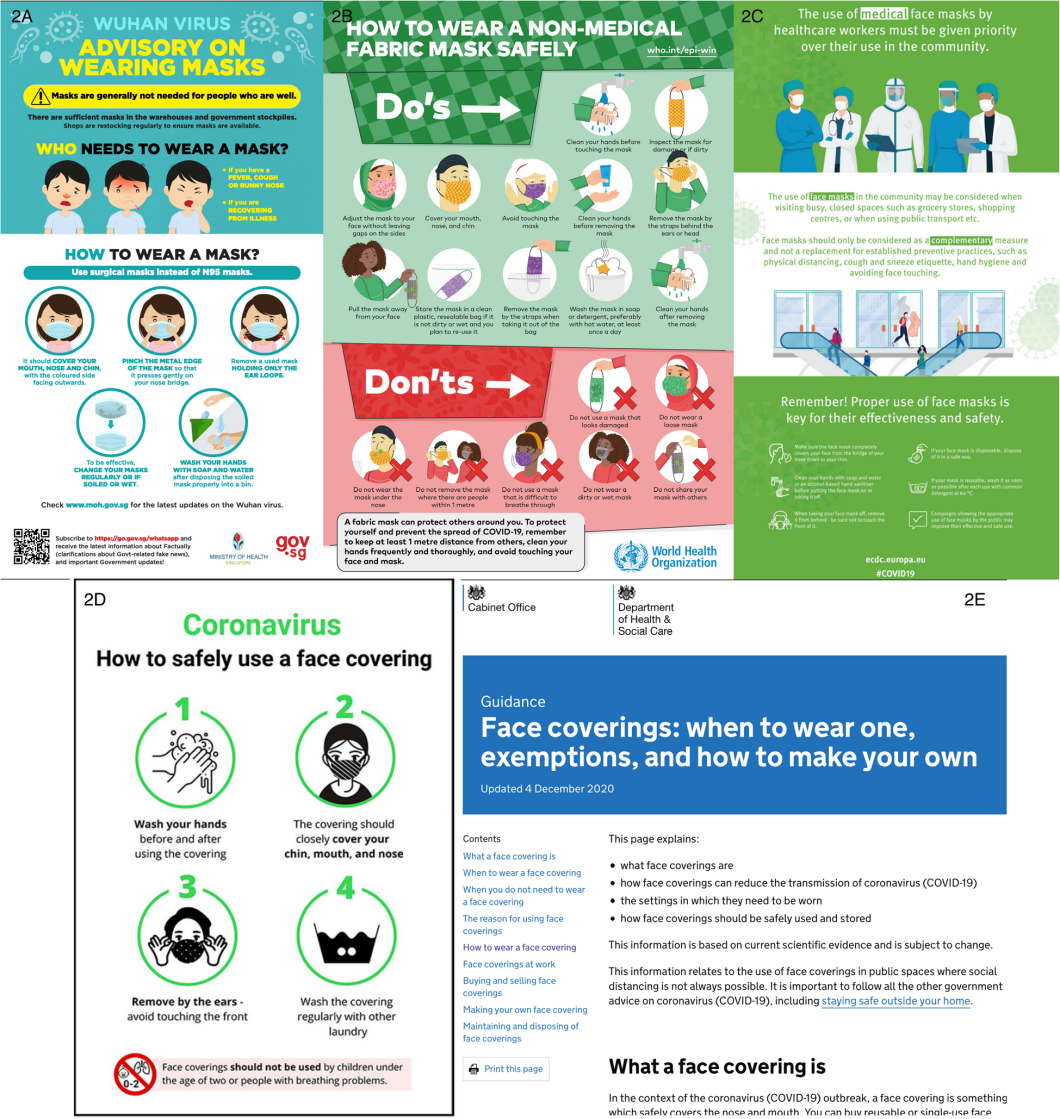
Excerpts of the main stimuli shown to each of the groups. The full stimuli are available at the referenced locations. Controls were shown no infographic or guidance. Permissions from relevant organisations have been sought to reproduce the infographics. **a**) Infographic from the SMOH [17]. **b**) Infographic from the WHO [18]. **c**) Infographic from the EUCDC [19]. **d**) Infographic from the BIT [20]. **e**) Text from gov.uk [21]

As this represented an evaluation of widespread public health guidance, all authors were not blinded to an individual’s allocation, however the author involved in the independent assignment of participants was not involved in data collection. The stimuli allocations included the (1) European Centre for Disease Prevention and Control (EUCDC) (2) World Health Organisation (WHO) guidance, (3) Singaporean Ministry of Health (SMOH), (4) a text-summary from the UK government (UK Gov) web-page and (5) a custom infographic designed by BIT based upon (4). The materials from the four infographics are shown in Fig. 2**.** The Singaporean Ministry of Health utilises a significant repository of COVID-related infographics which are freely available in the English language. This graphic was selected as a comparison from an Asian country in which face mask use is traditionally more prevalent. Furthermore, contrasts between the public health response to COVID measures in Singapore and the UK have been highlighted previously [22, 23].

These pictorial representations primarily focussed on how to correctly place a face mask from start to finish. They each highlight the desired behaviours of usage, with some providing additional information as to when use of a mask is not appropriate (e.g. for children under 2). The web-page UK government text outlined similar factual based information, although there were no graphical content, and no timeline. A control group were shown no stimuli, to provide a comparative baseline.

The Institutional Review Board was consulted for this evaluation of existing materials.

### Outcome measures

After reviewing the stimuli/infographics, all participants were asked to complete a questionnaire designed specifically by this study group and conducted by an independent author. The questionnaire was used to elicit baseline demographics including age, gender and ethnicity, recent symptomology and vulnerability to COVID-19. In addition, it focussed upon three domains of interest: recall, sentiment and willingness. These were chosen as key determinants of correct face mask wearing. Recall was focussed upon verifying a respondent’s understanding of how to correctly use a face covering, as outlined by the guidance from gov.uk. This was measured using 9 items. For each item that participants answered correctly, determined by selecting a correct response or not selecting the incorrect ones, they were awarded 1 point. The maximum recall score achievable was nine. They were also surveyed regarding their opinion about the design they had seen including content clarity, trustworthiness and its impact upon COVID-19-related anxiety *(Sentiment)*. This was measured using 5 items. For each item that participants expressed a positive sentiment (answering “moderately” or “very” to items such as “feeling confident about how to use a mask”), they scored 1 point. The maximum sentiment score was five. Participants were also asked to report their likelihood to use a mask or face covering when they went out in the future (*Willingness)* on a 4-point Likert scale. This was measured from 1 (No, definitely not) to 4 (Yes, definitely). Scores were totalled within each domain and analysed. The full English questionnaire can be seen as a **Supplementary File.**

### Control measures

Demographic data on participants’ age, gender, ethnicity, education level and recent COVID-19 symptomology was also collected. We also measured people’s current mask-wearing behaviours, their perception of the efficacy of masks, and their compliance with other healthcare practices.

### Analysis

To investigate the effect of the infographics (or UK Government text) upon the recall, sentiment and willingness to wear a mask, a series of Ordinary Least Squares (OLS) regression analyses were undertaken. These compared the effects of the stimulus, as well as the demographic covariates upon the dependent outcome measures. The OLS models were specified as follows:

$$ {Outcome}_i=a+{\beta}_T{Treatment}_i+{\beta}_C Co\mathit{\operatorname{var}}{iates}_i+{\in}_i;{\in_i}_{\sim}^{IID}N\;\left(0,{\sigma}^2\right) $$

Where, *Outcome*_*i*_ varied by model, including recall score, sentiment score, and willingness to use a mask. *T*_*i*_ was a vector of treatment indicators, equal to 0 if participant is assigned to the control condition. *Covariates*_*i*_ were a matrix of covariates that composed of: age group (dummy variables for “18~39 years old”, “40~59 years old”, and “60 and above”), gender (dummy variables for “male”, “female”), ethnic group (dummy variables for “White”, “Asian”, “Black”, and “Mixed/Others”) and educational attainment (dummy variables for “with degree”, “no degree”). ϵi is an independent error term.

## Results

### Descriptive statistics

A total of 4099 adult participants were enrolled into the study. No significant differences were observed between the age, gender, ethnicity or educational level between study arms (Table 1).

**Table 1:** Sample characteristics of the enrolled population

Subgroups (N)	Treatment conditions						
	Total (4099)	Control (1009)	EU CDC (628)	WHO (526)	SMOH (639)	BIT (606)	UK Gov (661)
**Age group**							
18–39 years	46.7	46.6	48.6	45.3	47.6	46.9	45.5
40–59 years	31.6	31.7	30.1	33.5	30.7	31.5	32.1
≥ 60 years	21.7	21.7	21.3	21.2	21.8	21.6	22.4
**Education**							
Degree	25.2	23.2	26.3	26.8	25.7	25.6	25.3
No degree	72.8	74.6	72.0	70.9	72.1	72.3	73.8
Prefer not to say	1.9	2.2	1.8	2.3	2.2	2.1	0.9
**Sex**							
Men	51.2	50.3	50.6	53.4	53.1	51.2	49.5
Women	48.8	49.7	49.4	46.6	46.9	48.8	50.5
**Ethnicity**							
White	86.0	85.9	87.7	86.3	85.8	83.5	86.5
Black	3.0	3.4	2.5	2.9	3.1	3.1	2.6
Asian	7.0	6.9	6.2	5.4	8.0	8.9	6.2
Mixed/other	4.1	3.8	3.5	5.4	3.1	4.5	4.7

*Values refer to percentages of each group*

### Recall

Recall of the salient steps of effective mask wearing was consistently high across all six groups. The World Health Organisation (7.40, *P* < 0.001), Singaporean Ministry of Health (7.38), and BIT groups (7.34, P < 0.001) demonstrated significantly higher average recall scores compared to controls, see Tables 2 & 3. Those who saw the UK government text advice had the same recall score as those who were given no visual stimulus (6.93 vs. 6.97). There was also substantial variance in recalling each individual item. Whereas 87.0% of the total cohort recalled that masks *should cover the nose*, only 53.1% recalled that it *should cover the chin. The recall was particularly poor among participants that* read the UK government text advice — 21% fewer participants recalling the covering-chin principle in the UK government text group compared to the BIT group, which was a visual representation of the same guidance. Similar patterns (inferior performance of the text advice) could be observed in all other trial arms. Of those who saw no guidance, a non-negligible minority held some potentially dangerous beliefs about how to use a mask —13.9% falsely believed that masks should be *loose enough to allow air in at the sides,* and 16.3% believed they should *pull down the mask if they need to cough*.

**Table 2:** Recall, sentiment and willingness scores of each of the visual stimuli groups

Subgroups (N)	Treatment conditions						
	Total (4099)	Control (1009)	EU CDC (628)	WHO (526)	SMOH (639)	BIT (606)	UK Gov (661)
**Recall items**							
Should cover nose	87.0	85.3	86.2	84.7	90.9	87.5	87.3
Should cover chin	53.1	50.9	54.0	52.5	64.3	60.4	38.7
Take off by ears	66.3	65.2	62.3	71.4	74.5	73.8	52.5
Avoid touching front after putting on	69.5	67.8	71.5	80.9	59.6	65.3	72.5
Wash hands if using	71.2	67.9	69.6	77.3	70.0	75.3	70.2
Should cover the neck	4.2	5.7	4.6	4.0	2.8	4.0	3.6
Should cover the forehead	4.2	5.8	5.6	3.8	2.0	3.8	3.5
Should fit loosely	11.5	13.9	10.5	10.3	10.3	10.1	12.1
Pull down mask to cough or sneeze	11.1	16.3	11.9	9.2	6.4	10.1	9.2
**Average recall score (SD)**	7.16 (1.62)	6.97 (1.69)	7.11 (1.69)	7.40 (1.61)	7.38 (1.45)	7.34 (1.59)	6.93 (1.55)
**Sentiment items**							
Easy to understand	87.3	NA	85.2	86.7	91.7	89.9	83.1
Trustworthy	85.5	NA	85.0	86.3	90.3	86.5	79.7
Confidence about mask-wearing	83.3	NA	80.4	84.4	85.8	86.8	79.7
Less anxious about coronavirus	49.6	NA	48.3	50.4	50.4	51.5	47.5
Right amount of information	87.5	NA	88.0	83.0	90.0	92.0	85.0
**Average sentiment score (SD)**	3.93 (1.24)	NA	3.86 (1.26)	3.91 (1.31)	4.08 (1.13)	4.06 (1.13)	3.75 (1.33)
**Willingness** Would use masks	87.0	88.2	86.0	88.9	86.7	87.1	84.4

*Values refer to percentages of each group*. *NA* not applicable*.* For recall items, participants were awarded one point if they answered “yes” for item 1 ~ 5, and “no” for item 6 ~ 9

**Table 3:** Results of the OLS regression showing β coefficients adjusted for covariates for recall scores, sentiment scores, and willingness to use a mask for each infographic group

	*Outcome Measures*					
	Recall Score		Sentiment Score		Willingness to Use a Mask	
	β	95% CI	β	95% CI	β	95% CI
UK Gov	−0.061	−0.212, 0.090	Ref	Ref	−0.037^*^	--0.070, −0.004
EU CDC	0.140	−0.013, 0.294	0.120	−0.014, 0.255	−0.022	−0.056, 0.011
WHO	0.436^***^	0.277, 0.596	0.168^*^	0.029, 0.307	0.008	−0.026, 0.043
SMOH	0.432^***^	0.279, 0.584	0.333^***^	0.199, 0.467	−0.016	− 0.049, 0.017
BIT	0.400^***^	0.245, 0.555	0.315^***^	0.179, 0.450	−0.012	−0.046, 0.021
Gender: Male	−0.461^***^	− 0.556, − 0.366	−0.072	− 0.159, 0.016	−0.020	− 0.041, 0.0003
Age Group: 40–59	0.496^***^	0.386, 0.606	0.156^**^	0.055, 0.258	−0.041^***^	− 0.065, − 0.017
Age Group: 60+	0.710^***^	0.585, 0.836	0.225^***^	0.110, 0.341	−0.024	− 0.051, 0.003
Ethnicity: Asian	− 0.754^***^	− 0.943, − 0.565	0.106	− 0.069, 0.280	0.070^***^	0.029, 0.111
Ethnicity: Black	−0.529^***^	− 0.810, − 0.249	0.182	− 0.082, 0.446	0.043	− 0.018, 0.104
Ethnicity: Mixed/other	−0.577^***^	− 0.816, − 0.337	−0.182	− 0.400, 0.036	0.006	− 0.046, 0.058
Education: No degree	−0.022	− 0.131, 0.087	0.005	− 0.095, 0.105	−0.013	− 0.037, 0.011
Constant	7.010^***^	6.860, 7.150	3.680^***^	3.540, 3.820	0.914^***^	0.882, 0.946
Observations	4099	4099	3090	3090	4099	4099
R^2^	0.015	0.099	0.010	0.019	0.002	0.010
Adjusted R^2^	0.013	0.096	0.009	0.015	0.001	0.007
Residual Std. Error	1.610	1.540	1.240	1.230	0.337	0.336
F Statistic	12.200^***^	34.500^***^	8.070^***^	4.920^***^	1.500	3.210^***^

^*^p^**^p^***^p < 0.001

*Note 1:* The reference group for recall score and willingness to use a mask was the no-stimuli control condition. The reference group for sentiment score was UK government’s text guidance, thus the number of observations was smaller than that of the other outcome measures. The reference age group was 18–39 years old, the reference ethnicity group is White, and the reference education group is with degree

*Note 2*: Adjusting for covariates did not affect the sign and significance level compared to non-adjusted analysis (not shown)

### Sentiment

Participant average sentiment scores were again significantly higher in those who had seen the Singaporean MOH (4.08, *P* < 0.001), the BIT (4.06, P < 0.001), and the WHO stimuli (3.91, *P* < 0.05), compared controls see Tables 2 & 3. In particular, participants were more likely to consider the mask-wearing guidance as easy to understand and felt more confident about how to wear a mask properly. All 5 groups tested (control group was not asked about sentiment-related questions) were equally ineffective in reducing anxiety level, with only 48.3% of the total cohort reporting less anxious about getting coronavirus after reading the stimuli. Participants that read the UK government text advice were least likely to feel the mask-wearing guidance was *trustworthy* or felt *confident about mask-wearing*.

### Willingness

The willingness of using a mask in the future was high among all participants (87%). However, participants that read the UK government’s text guidance were less (3.8, 95% CI [0.4, 7.0%]) willing to do so compared with those that did not read any stimuli. The willingness to use a mask was comparable among the rest of the trial arms. We also observed that Asian were more willingness than White respondents to use a mask (7.0, 95% CI [2.9, 11.1%]), see Table 3.

### Secondary measures

**Fig. 3:**
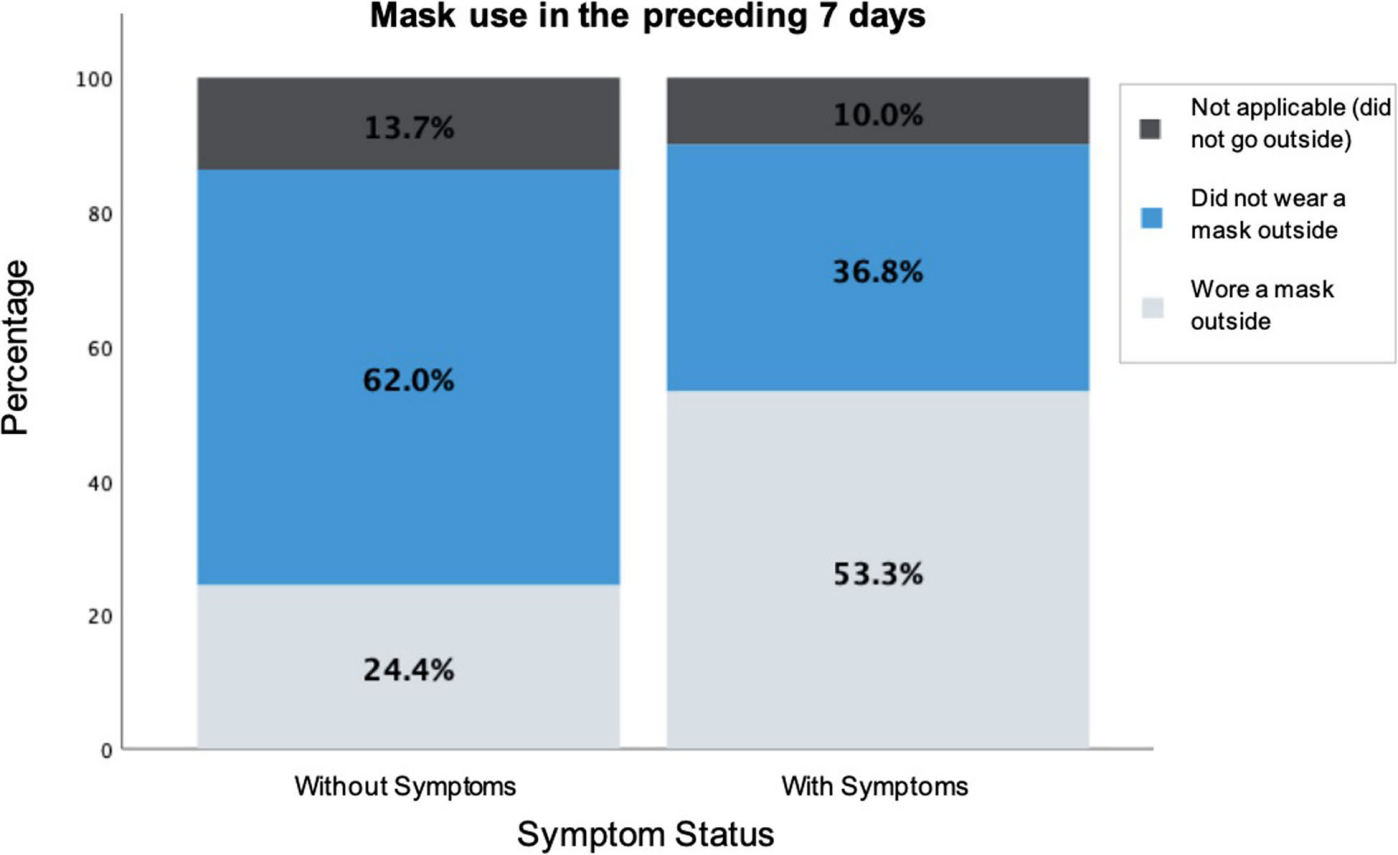
Mask use in the 7 days prior to the study by symptom status. *With Symptoms* refers to the presence of fever, cough or anosmia

A total of 262 respondents reported one of three COVID-19 symptoms within the preceding 7 days of the study (fever, cough, anosmia). Of those with symptoms 53.3% reported wearing a face mask when outside (Fig. 3). Furthermore, those with symptoms were significantly less likely to comply with handwashing (mean days hand washed 4.9 v. 6.3, *p* < .05) and social distancing (means days distanced 4.7 v. 5.7, *p* < .05) compared to those without symptoms.

## Discussion

This evaluation demonstrates the impact of differing visual stimuli and guidance, upon the recall of technique, sentiment and willingness of using a face mask in a UK based cohort. We have shown that three infographics developed by the Behavioural Insights Team, the Singaporean Ministry of Health and the World Health Organisation led to a higher overall recall of the appropriate techniques in using a mask. Moreover, this study has highlighted that whilst 85.2% of the cohort felt the information was effectively represented, the impact upon COVID-19 fear is low, with less than half of respondents reporting lower anxiety. Despite this, the willingness for participants to use a mask was consistently high across the groups, ranging from 84 to 88%.

Our results are in keeping with recent trends of increasing support for the use of face masks in public [24]. The estimation that 40% of COVID-19 transmission is by asymptomatic individuals, has led many to advocate for the recent UK legislation [25]. Whilst definitive data is lacking with respect the effectiveness of masks in isolation, potentially explaining the low anxiety-relief of stimuli, mask use is likely to be of greater benefit than no covering at all [26]. This has led to the suggestion of a social norm of wearing a mask [27]. Increasing their use alone, however, is insufficient, as members of the public require the knowledge to use the coverings appropriately. Lee et al. demonstrated that in a pre-pandemic population, none of the 1500 adults enrolled in their study, correctly completed all the necessary steps to apply a face mask. Furthermore, only 8.5% performed adequate hand hygiene when using the mask [28]. We have demonstrated similar results, with just 35.5% of respondents recalling to wash their hands when using masks. Those who were exposed to infographics, however, did outperform the other groups in this respect.

The use of infographics has been demonstrated to improve health information transfer and knowledge, in areas such as dementia care and hand hygiene [29, 30]. A previous study by BIT, showed over 90% of respondents reported better understanding of handwashing technique after reviewing a poster [31]. In the current study, we have demonstrated that infographics helped increase knowledge compared to controls. In fact, 33% fewer respondents in the BIT group reported they would perform potentially dangerous behaviours, such as removing their masks to cough, compared to those who had no guidance. Furthermore, the use of infographics helped to reduce cognitive burden, as has been seen in other studies [32]. This was evidenced by the higher recall scores achieved by the BIT compared UK government text, despite being based upon the same content. This differentiation may also be explained by the provenance of the information, as trustworthiness and confidence about mask wearing were lower in the government group by 6 and 7%, respectively [33]. Cognitive burden may also explain the differences seen between the three infographics. BIT and SMOH utilised a simple cartoon to increase the salience of appropriate mask technique. On the other hand, the WHO dichotomised the process into desirable and undesirable behaviours (‘Do’s and Don’ts). 9% fewer respondents however felt the information amount was correct compared to the BIT cohort.

The findings from this study have significant health policy implications. A common criticism of COVID-19-related guidance has been a lack of clarity regarding preventative measures [34]. We have shown by developing custom visual aids, dissemination of health information can be effectively undertaken to a population representative that of the UK. This is particularly important when attempting to overcome the several barriers to the use of face masks that have been suggested; including a lack of their perceived benefit [35], a lack of perceived susceptibility [36], and the un-enforceability of the law [37]. Such information can also be tailored to appreciate differing cultural values. For example, there is a predilection to reserve masks for usage when ill in Western countries, as oppose ubiquitously as prevention in Asia [38]. This is further highlighted by the fact that, concerningly, whilst only 53.3% of symptomatic participants reported using a face mask in the preceding week, this was still more than double the asymptomatic group (24.4%). When presenting public health information via infographics such social values can be acknowledged.

Although we utilised a randomised methodology, in a cohort that represents the UK population, and enrolled a large number of participants, there were limitations with this study. Firstly, there was a potential for selection bias, as the study was conducted online and those with poorer digital literacy may have not enrolled. This was appropriate as we conducted this research within the constraints of social distancing measures, which have also favoured the utilisation of electronic resources generally [39]. Secondly, we used *willingness* to wear a mask as an outcome, as oppose to actual mask usage. We acknowledge the difference between contemplation and action of health behaviours [40]. This may explain while in the current study 87% reported a willingness to wear a mask, only 18% reported wearing a mask on a YouGov poll at the same time [7]. To mitigate this, we contrasted the rates of willingness, amongst those who had high and low use of a mask in the previous 7 days and elicited similar findings.

Future work will need to address these limitations, and examine the impact of visual stimuli on actual utilisation of masks. In addition, as no modality led to 100% recall or feedback, we aim to develop the BIT tool further. By means of an iterative process, using participant feedback, we hope to be able to disseminate clear, robust information to encourage the future use of face masks in the midst of the COVID-19 recovery.

## Conclusion

Visual stimuli in the form of infographics have the capacity to improve the public’s compliance, as well as their technique, with precautionary measures, such as face masks. This is integral in the ongoing effort to reduce the transmission of COVID-19, and its significant public health effects. Work, however, is needed to develop these tools in several aspects, including the effect they have on COVID-19-related anxiety.

## Supplementary Information

**Additional file 1.**

## Data Availability

The data that support the findings of this study are available from The Behavioural Insights Team but restrictions apply to the availability of these data, which were used under license for the current study, and so are not publicly available. Data are however available from the authors upon reasonable request and with permission of The Behavioural Insights Team/Imperial College London.
