# Supplementary material for: Evaluating the effect of infographics on public recall, sentiment and willingness to use face masks during the COVID-19 pandemic: a randomised internet-based questionnaire study
Source: BMC Public Health. 2021 Feb 17;21:367. doi: 10.1186/s12889-021-10356-0 (PMC7886844; doi:10.1186/s12889-021-10356-0)
Supplement: Supplementary file 1 — Additional file 1. [file 12889_2021_10356_MOESM1_ESM.docx]

**Additional File 1**

*All responses appeared in random order. Red* represents incorrect response and *green* represents correct response.

**Recall**

**After seeing the guidance, which of the following do you think are recommended when using a facemask or face covering? Please tick all that apply**

- Mask should cover the nose
- Mask should cover the chin
- Take off mask using the ear loops
- Wash hands if using a mask
- Avoid touching the front after you’ve put it on
- Mask should cover the neck
- Mask should cover the forehead
- Mask should fit loosely and allow in air at the sides
- Pull down mask if you need to cough or sneeze

**After seeing the guidance, which of the following groups do you think are not recommended to wear a facemask or face covering? Tick all that apply**

- Children age 0-2
- People with breathing problems
- Pregnant women
- People aged 70+
- People who have coronavirus symptoms
- Children age 5-11

**Confidence and Intent**

**After seeing the guidance, would you be willing to use a face mask or face covering?**

- Yes definitely

- Yes probably

- No probably not

- No definitely not

**If not, why would you not be willing to wear a facemask or face covering when you go outside? Tick all that apply**

- I am not in the habit of using them
- I think only sick people should wear them
- I do not want to wear them if most other people are not wearing them
- I do not want to wear them because I do not think they are effective
- I do not want to wear one for another reason (please specify)

**If you would not be willing to wear a mask, which of the following do you think would make you feel more willing to wear a facemask or face covering? Tick all that apply**

- If the government recommended that people should use them
- If the NHS recommended that people should use them
- If I saw many other people wearing them outside
- Other, please specify
- None of the above would make me feel willing to wear a face mask or face covering

**After seeing the guidance, do you intend to wear a face mask or face covering when you...**

- take public transport (yes / no / does not apply)

- go to the shops (yes / no / does not apply)
- go outside for exercise (yes / no / does not apply)
- go to work (yes / no / does not apply)
- are at home (yes / no)

**Sentiment**

*Participants are shown the original poster again while answering these questions*

**Looking at the guidance again, would you say it**

- is easy to understand
- is trustworthy
- makes me feel more confident that I know how to use a facemask or face covering
- makes me feel less anxious about the risk of coronavirus

[Not at all / A little / Moderately / Very]

**Would you say the guidance has:**

- too little information

- about the right amount of information
- too much information

**Do you have any other feedback about the guidance?**

[free text box]

**Current mask use, symptoms and attitudes**

**In the last 7 days, when you went outside for more than a few minutes, did you usually wear a facemask or face covering?**

- Yes
- No
- Does not apply because I did not go outside in the last 7 days

**Over the last 7 days, on how many different days (0-7 days) did you...**

- wash your hands for 20 seconds with soap and water or hand sanitiser
- consistently keep at least 2 metres away from people who do not live with you when you go outside

**Would you wear a....**

- medical mask
- homemade mask
- fabric mask
- scarf or bandana

**Do you think that wearing facemasks or face coverings...**

- Helps reduce your risk of catching coronavirus
- Helps reduce your risk of spreading coronavirus to others
- Both of the above
- None of the above

**If most people in the UK wore facemasks or face coverings, to what extent do you think this would help reduce the spread of coronavirus?**

[Not at all / Slightly / Moderately / Very]

**If you saw someone in a crowded place, like a shop or public transport, who was not wearing a mask or face covering, what word or phrase would come to mind about that person?**

[free text feedback]

**In the last 7 days, did you have any of the following symptoms? Tick all that apply**

- new continuous cough
- fever
- sneezing
- runny nose
- diarrhoea
- nausea or vomiting
- loss of appetite
- loss of sense of smell
- none of the above

Are you classed as vulnerable?

[Yes/No/Don’t Know]

Vulnerable means you:

- Are pregnant
- Are over the age of 70
- Have an Underlying health condition
